# Donor support for quality assurance and pharmacovigilance of anti-malarials in malaria-endemic countries

**DOI:** 10.1186/s12936-017-1921-x

**Published:** 2017-07-11

**Authors:** Stephanie D. Kovacs, Brianna M. Mills, Andy Stergachis

**Affiliations:** 1 Department of Epidemiology, University of Washington, 6123 SW Stevens Street Apt 302, Seattle, WA 98116 USA; 2 Center for Studies in Demography and Ecology, University of Washington, Seattle, WA USA; 3 Department of Global Health, University of Washington, Seattle, WA USA; 4 Department of Pharmacy, University of Washington, Seattle, WA USA

**Keywords:** Malaria, Pharmacovigilance, Adverse events, Health system strengthening, Artemisinin-based combination therapy

## Abstract

**Background:**

Malaria control efforts have been strengthened by funding from donor groups and government agencies. The Global Fund to Fight AIDS, Tuberculosis and the Malaria (Global Fund), the US President’s Malaria Initiative (PMI) account for the majority of donor support for malaria control and prevention efforts. Pharmacovigilance (PV), which encompasses all activities relating to the detection, assessment, understanding, and prevention of adverse effects or any other drug-related problem, is a necessary part of efforts to reduce drug resistance and improve treatment outcomes. This paper reports on an analysis of PV plans in the Global Fund and PMI and World Bank’s grants for malaria prevention and control.

**Methods:**

All active malaria grants as of September 2015 funded by the Global Fund and World Bank, and fiscal year 2015 and 2016 PMI Malaria Operational Plans (MOP) were identified. The total amount awarded for PV-related activities and drug quality assurance was abstracted. A Key-Word-in-Context (KWIC) analysis was conducted for the content of each grant. Specific search terms consisted of pharmacovigilance, pregn*, registry, safety, adverse drug, mass drug administration, primaquine, counterfeit, sub-standard, and falsified. Grants that mentioned PV activities identified in the KWIC search, listed PV in their budgets, or included the keywords: counterfeit, sub-standard, falsified, mass drug administration, or adverse event were thematically coded using Dedoose software version 7.0.

**Results:**

The search identified 159 active malaria grants including 107 Global Fund grants, 39 fiscal year 2015 and 2016 PMI grants and 13 World Bank grants. These grants were primarily awarded to low-income countries (57.2%) and in sub-Saharan Africa (SSA) (70.4%). Thirty-seven (23.3%) grants included a budget line for PV- or drug quality assurance–related activities, including 21 PMI grants and 16 Global Fund grants. Only 23 (14.5%) grants directly mentioned PV. The primary focus area was improving drug quality monitoring, especially among the PMI grants.

**Conclusions:**

The results of the analysis demonstrate that funding for PV has not been sufficiently prioritized by either the key malaria donor organizations or by the recipient countries, as reflected in their grant proposal submissions and MOPs.

**Electronic supplementary material:**

The online version of this article (doi:10.1186/s12936-017-1921-x) contains supplementary material, which is available to authorized users.

## Background

Malaria is a major cause of morbidity and mortality with 3.6 billion people living in areas of malaria transmission [1]. In 2015, there were 416 million cases of malaria resulting in 438,000 deaths [1], the majority of which occurred in sub-Saharan Africa (SSA). Artemisinin-based combination therapy (ACT) is the most widely used anti-malarial therapy. According to WHO, 337 million ACT treatment courses were delivered to both the public and the private sectors in endemic countries in 2014 [2]. Malaria control efforts have been strengthened by funding from donor groups and government agencies, especially the Global Fund to Fight AIDS, Tuberculosis and Malaria (Global Fund) and the US President’s Malaria Initiative (PMI) which account for the majority of donor support for malaria control efforts. According to a 2013 report by the Kaiser Family Foundation, the Global Fund and PMI are the two largest donors for malaria assistance representing 83% of all global assistance [3]. In 2015, PMI and the Global Fund invested 861 million USD and 8.075 billion USD, respectively, in malaria control programmes around the world [3, 4].

While anti-malarials are generally considered safe and well tolerated, potential safety concerns warrant strengthening their pharmacovigilance in malaria-endemic countries. For example, there have been numerous calls for systematically monitoring the safety of ACT during pregnancy through pregnancy exposure registries [5, 6]. This is due to non-clinical findings of adverse effects of artemisinins on early development of the fetus seen only in animal studies [7, 8]. Moreover, small observational studies have reported an association between the repeated dosing of dihydroartemisinin–piperaquine and prolongation of the QT interval [9, 10]. Additionally, there are limited data on the safety of primaquine, given its known association with haemolytic anaemia in patients who are deficient in glucose-6-phosphate dehydrogenase (G6PD) [11]. Furthermore, artesunate/amodiaquine has been associated with acute extrapyramidal reactions is both children and adults [12]. Moreover, there is low tolerability and associated poor compliance with the use of quinine [13, 14]. With regard to product quality, studies have shown that up to 35% of anti-malarial drugs in SSA and Southeast Asia failed chemical analyses and additionally, 20% of anti-malarial drugs were found to be falsified [15]. Falsified, counterfeit and sub-standard anti-malarial drugs pose a great health risk as they can lead to sub-optimal treatment and promote drug resistance [13].

Pharmacovigilance (PV), which encompasses all activities relating to the detection, assessment, understanding, and prevention of adverse effects or any other drug-related problem, is a necessary part of efforts to reduce drug resistance and improve treatment outcomes [16]. PV activities include spontaneous and active surveillance systems for detecting drug-related adverse events, pregnancy exposure registries, laboratory support to detect and prevent sub-standard and falsified pharmaceuticals, and improvements to the regulatory system. A growing number of low- and middle-income countries (LMICs) have some form of a functional PV system. Donor support has contributed to strengthening the capacity of LMICs to conduct PV [17]. However, many LMICs lack some or all of WHO’s basic elements of a PV system [18] and rely largely on spontaneous adverse event reporting systems [19]. Spontaneous reporting rates of adverse drug reactions in LMICs, including those associated with anti-malarials in SSA, are very low [20, 21]. Other important gaps in LMICs’ national PV programmes have been cited, including lack of funding and the need to strengthen training of those involved in PV [22].

This paper is an update of a situational analysis of PV plans included in the Global Fund Malaria and PMI-funded proposals first conducted in 2010 [23]. Since publication, there has been an expansion of malaria eradication efforts, increase of use of ACT, and changes to the Global Fund’s grant-making procedures. The goal of this paper is to assess PV efforts in the context of these developments.

## Methods

**Fig. 1:**
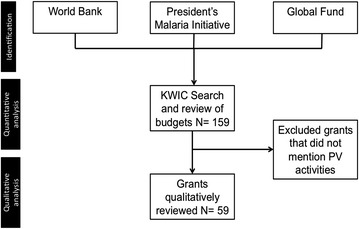
Data flow diagram

This study identified all active malaria grants as of September 2015 funded by the Global Fund and the World Bank, as well as active grants for fiscal year 2015 and 2016 Malaria Operational Plans (MOP) for PMI. For each active grant, the total amount awarded and the total amount allocated for PV- and drug quality assurance-related activities within the approved budgets was abstracted. A Key-Word-in-Context (KWIC) analysis was conducted for the content of each grant. Specific search terms consisted of pharmacovigilance, pregn*, registry, safety, adverse drug, mass drug administration, primaquine, counterfeit, sub-standard, and falsified (Fig. 1).

Grants that mentioned PV activities and/or drug quality assurance identified in the KWIC search, listed these terms in their budgets, or included the keywords: counterfeit, sub-standard, falsified, mass drug administration, or adverse event, were thematically coded using Dedoose software version 7.0.23, web application for managing, analysing, and presenting qualitative and mixed method research data (Socio Culture Research Consultants LLC) (N = 59 grants). The list of thematic codes was developed a priori to categorize activities described in the grants using definitions developed by WHO [24, 25]. The codes included: pharmacovigilance, supply chain management, mass drug administration, public health surveillance, malaria in pregnancy, and health system strengthening. PV was further divided into three types: detecting counterfeit drugs, drug quality assurance and detecting adverse events (for the complete code book, see Additional file 1).

After excerpts were thematically categorized, they were coded by the following activity types based on activity descriptions in the grants: behaviour change communication, improvement of regulatory system, supply chain management, laboratory support and training, operational research, or surveillance, training/capacity building. Excerpts with insufficient detail of the activities were categorized as activity not otherwise specified. PV-related excerpts were reviewed and grouped by geographic region and grant-making organization to identify additional themes. For the quantitative data, the study reported numbers and percent or mean and range as appropriate, stratified by grant-making organization.

## Results

### Identification and categorization of pharmacovigilance and quality assurance activities

**Fig. 2:**
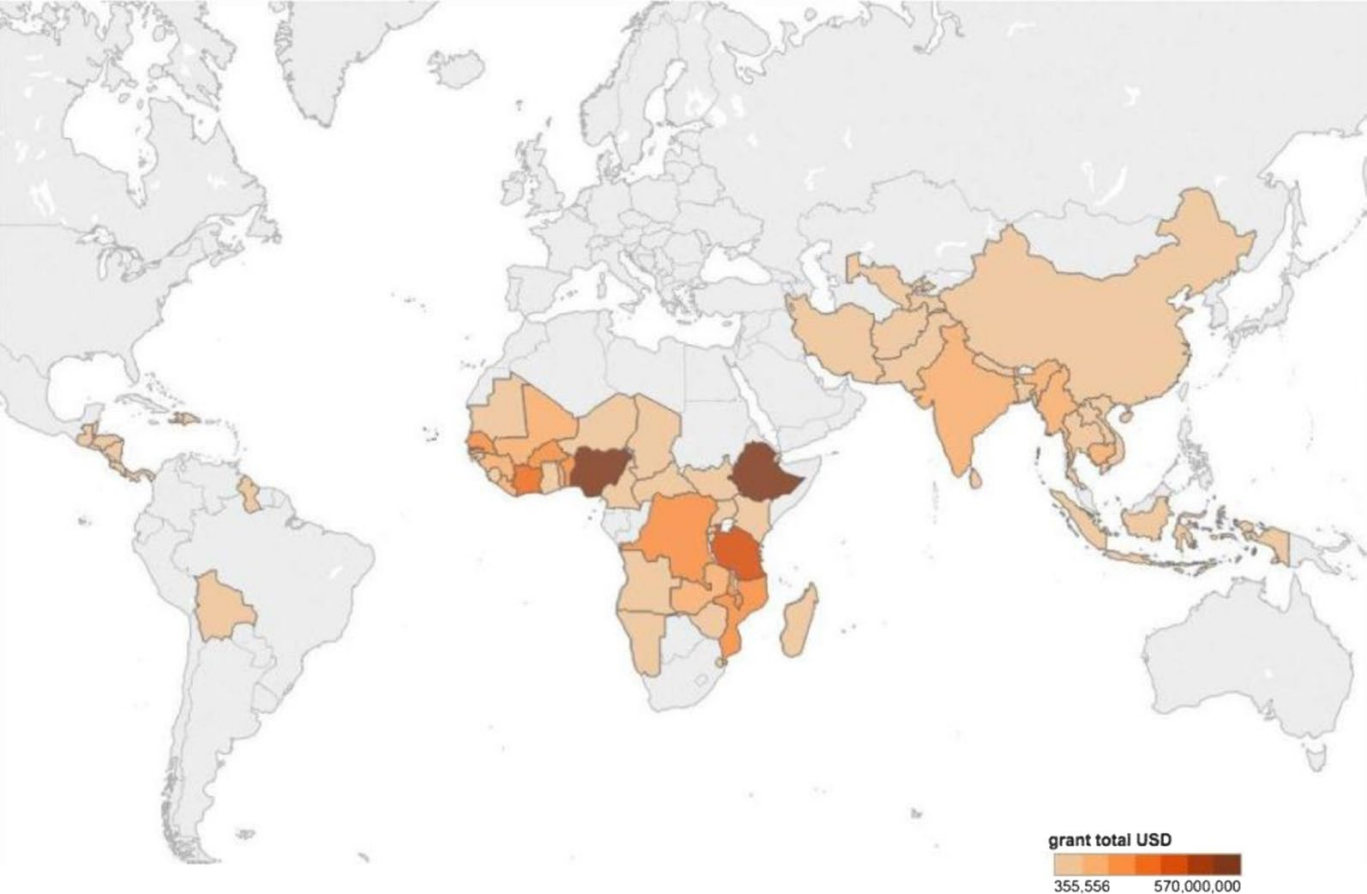
Location of malaria grants funded by PMI, Global Fund, and the World Bank

**Fig. 3:**
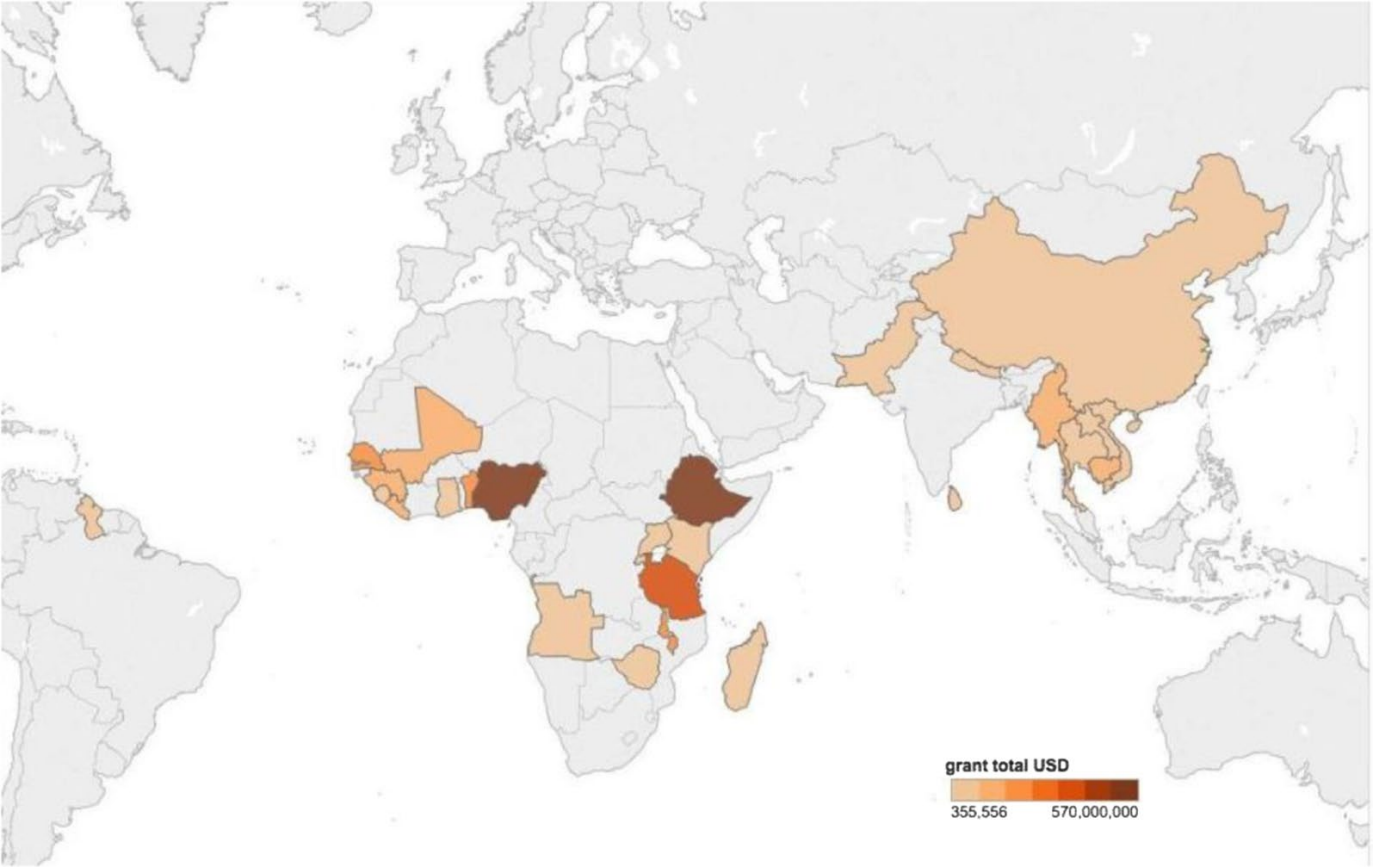
Location of malaria grants that described pharmacovigilance activities

**Fig. 4:**
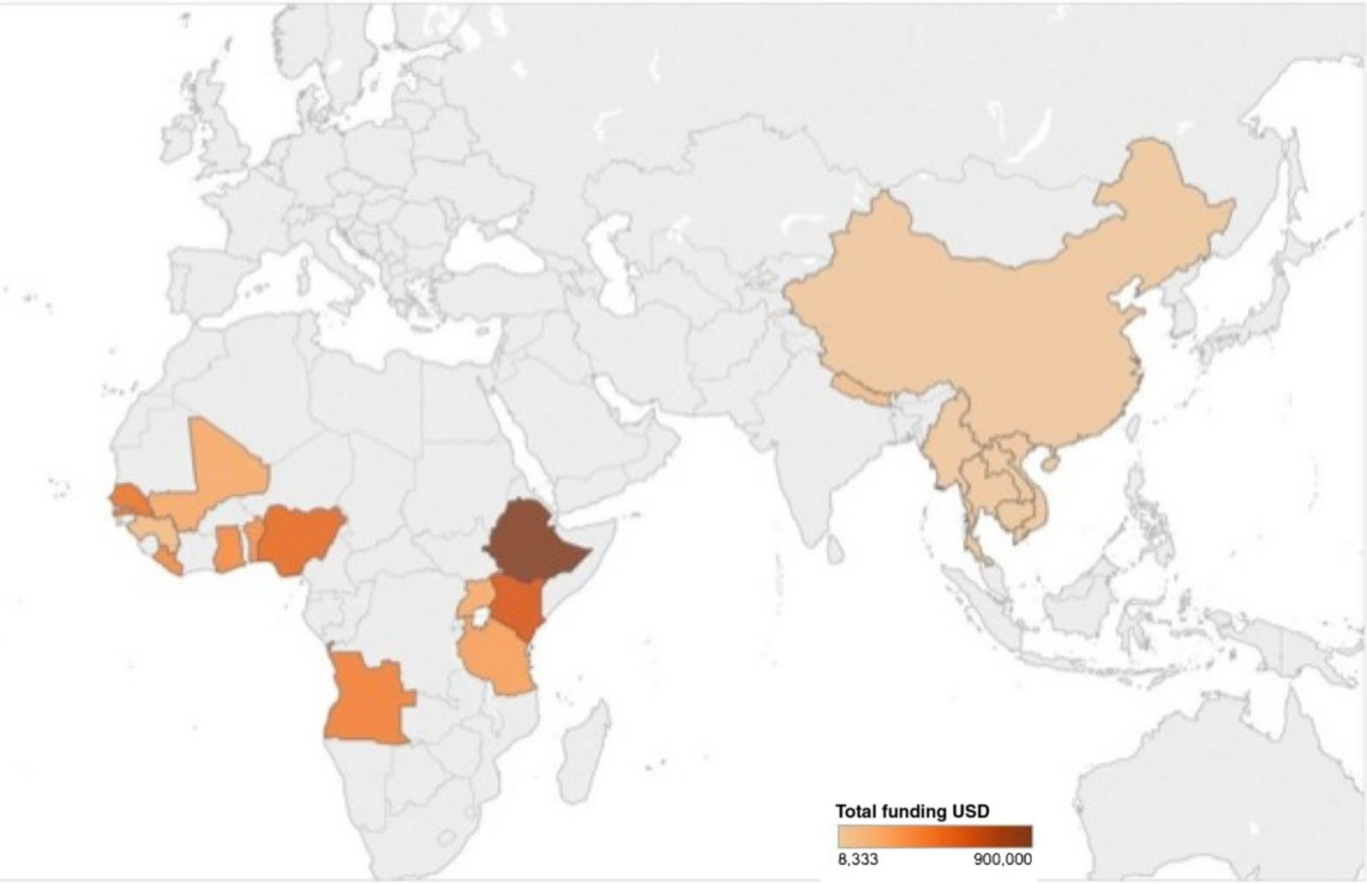
Locations of grants that directly funded pharmacovigilance in their budgets

The search identified 159 active malaria grants, including 107 Global Fund grants, 39 fiscal year 2015 and 2016 PMI grants, and 13 World Bank grants for review. These grants were primarily awarded to recipients in low-income countries (57.2%) and SSA (70.4%) (Fig. 2). The mean grant size was 33.3 million USD (range 0.6–500.0 million USD). Thirty-seven (23.3%) grants provided a budget line for PV activities, including 21 PMI grants and 16 Global Fund grants. Only 23 (14.5%) grants directly mentioned PV. During the qualitative coding of the data, 28% of grants described activities that were determined to be related to PV (Fig. 3). On average, grants that funded PV activities allocated 211,000 USD (range 31,000–500,000 USD) per grant for PV (Fig. 4). Although no grants funded pregnancy registries and only five (3%) mentioned them, 75 (47.2%) grants funded malaria in pregnancy activities, 97 (61.0%) funded anti-malarial drug procurement, and 38 (23.9%) funded some form of surveillance (Table 1).

**Table 1:** Description of active malaria grants, stratified by grant-making organization

	PMI, N = 39, N (%)	Global Fund N = 107, N (%)	World Bank N = 13, N (%)	Total, N = 159, N (%)
WHO Region				
Americas	0 (0)	8 (7.5)	0 (0)	8 (5.0)
Africa	37 (94.9)	64	11	
Eastern Mediterranean	0 (0)	1 (0.9)	1 (7.7)	2 (1.3)
East Asia and Pacific	2 (5.1)	16 (15.0)	1 (7.7)	19 (12.0)
South Asia	0 (0)	13 (12.2)	0 (0)	13 (8.2)
Europe	0 (0)	5 (4.7)	0 (0)	5 (3.1)
World Bank income group				
Upper-middle income	4 (10.3)	8 (7.5)	0 (0)	12 (7.6%)
Lower-middle income	8 (20.5)	45 (42.1)	3 (23.1)	56 (35.2%)
Low income	27 (69.2)	54 (50.5)	10 (76.9)	91 (57.2%)
Amount of grant^a^	12.7 million USD (3–70 million USD)	30.0 million USD (0.6–284 million USD)	82.0 million USD (46–500 million USD)	33.3 million USD (0.6–500 million USD)
Pharmacovigilance mentions				
Grant mentions pharmacovigilance	21 (53.9)	2 (1.9)	0 (0)	23 (14.5)
Grant provides budget line for pharmacovigilance	21 (53.9)	16 (15.0)	0 (0)	37 (23.3)

^a^Denotes a mean and range

The study identified 288 excerpts in 59 grants that described PV-related activities, i.e., 36 PMI grants and 23 Global Fund grants. No World Bank grants described PV activities and were excluded from data abstraction. The plurality of these excerpts provided context for grant implementation (n = 39) or described historical activities (n = 69) rather than describing a current grant-supported activity. The most common PV-related activities described were laboratory support and training (n = 39), improving the regulatory system (n = 32), surveillance (N = 36), and PV activities not otherwise specified (NOS) (n = 32). The most common PV area of interest was drug quality assurance (n = 154), and only 14 grant excerpts described activities to detect adverse events (Table 2).

**Table 2:** Count of activities mentioned in pharmacovigilance-related coded excerpts, stratified by type (N = 288)

Pharmacovigilance area of interest						
Activity type	Adverse events	Counterfeit drugs	Drug quality assurance	PV NOS	N/A	Total
Behaviour change communication		6 (85.7)	1 (14.3)			7 (2.4)
Improve regulatory system	1 (3.1)	6 (18.8)	14 (53.1)	11 (34.4)		32 (11.1)
Improving supply chain		1 (6.7)	7 (46.7)	7 (46.7)		15 (5.2)
Laboratory supplies and training		6 (15.4)	27 (69.2)	6 (15.4)		39 (13.5)
Operations research	2 (66.7)		1 (33.3)			3 (1.0)
Surveillance	3 (8.3)	10 (27.8)	21 (58.3)	2 (5.6)		36 (12.5)
Training/capacity building		1 (9.1)	6 (54.5)	4 (36.4)		11 (3.8)
Pharmacovigilance NOS	2 (6.3)	1 (3.1)	23 (71.9)	6 (18.8)		32 (11.1)
Pharmacovigilance NOS-unfunded			26 (100)			26 (9.0)
Surveillance-not funded	5 (100)					5 (1.7)
Context only^a^					39 (100)	39 (13.5)
Historical activity^b^		12 (17.4)	37 (53.6)	17 (24.6)	3 (4.4)	69 (24.0)
Total	14 (4.9)	40 (13.9)	154 (53.5)	49 (17.0)	42 (14.6)	

^a^Excerpts that described pharmacovigilance activities occurring within the country but that were not supported or funded by the current or past grants

^b^Excerpts that described pharmacovigilance activities that were previously funded by the grant making organization but were not being supported in the current grant

The majority of grant excerpts were from grants in SSA (65.3%). Grants to SSA countries were more likely to describe PV activities to improve the regulatory system than grants to Asian countries (13.8 vs 6.3%, respectively) and less likely to describe surveillance activities (11.2 vs 16.8%, respectively). Grants to Asian countries were more likely to describe unfunded PV activities, i.e., grants that described activities but provided no funding in the budget, than grants to SSA (17.9 vs 3.2%) (Tables 3, 4).

**Table 3:** Count of activities mentioned in pharmacovigilance excerpts from Africa, stratified by type (N = 188)

Pharmacovigilance area of interest						
Activity type	Adverse events	Counterfeit drugs	Drug quality assurance	PV NOS	NA	Total
Behaviour change communication		3 (75.0)	1 (25.0)			4 (2.1)
Improve regulatory system		5 (19.2)	12 (46.2)	9 (34.6)		26 (13.8)
Improving supply chain			5 (45.4)	6 (54.5)		11 (5.9)
Laboratory supplies and training		4 (18.2)	17 (77.3)	1 (4.5)		22 (11.7)
Operations research	2 (100)					2 (1.1)
Pharmacovigilance NOS	2 (8.7)	5 (21.7)	15 (65.2)	1 (4.3)		23 (12.2)
Surveillance	2 (9.5)	5 (23.8)	13 (61.9)	1 (4.8)		21 (11.2)
Training/capacity building			5 (55.6)	4 (44.4)		9 (4.8)
Surveillance-not funded	1 (100)					1 (0.5)
Pharmacovigilance NOS-unfunded			6 (100)			6 (3.2)
Context only^a^					35 (100)	35 (18.6)
Historical activity^b^		6 (13.3)	26 (57.8)	12 (26.7)	1 (3.8)	45 (23.9)
Total	7 (3.7)	22 (11.7)	94 (50.0)	35 (18.6)	36 (19.1)	188 (100.0)

^a^Excerpts that described pharmacovigilance activities occurring within the country but that were not supported or funded by the current or past grants

^b^Excerpts that described pharmacovigilance activities that were previously funded by the grant making organization but were not being supported in the current grant

**Table 4:** Count of activities mentioned in pharmacovigilance excerpts from Asia, stratified by type (N = 95)

Pharmacovigilance area of interest						
Activity type	Adverse events	Counterfeit drugs	Drug quality assurance	PV NOS	NA	Total
Behaviour change communication		3 (100)				3 (3.2)
Improve regulatory system	1 (16.7)	1 (16.7)	2 (33.3)	2 (33.3)		6 (6.3)
Improving supply chain		1 (25.0)	3 (75.0)			4 (4.2)
Laboratory supplies and training		2 (14.3)	7 (50.0)	5 (35.7)		14 (14.7)
Operations research			1 (100)			1 (1.1)
Pharmacovigilance NOS		1 (11.1)	8 (88.9)			9 (9.5)
Surveillance	1 (6.3)	4 (25.0)	10 (62.5)	1 (6.3)		16 (16.8)
Training/capacity building		1 (50.0)	1 (50.0)			2 (2.1)
Surveillance-not funded	3 (100)					3 (3.2)
Pharmacovigilance NOS-unfunded			17 (100)			17 (17.9)
Context only^a^					3 (100)	3 (3.2)
Historical activity^b^		6 (25.0)	11 (45.8)	5 (20.8)	2 (8.3)	24 (25.3)
Total	5 (5.3)	18 (18.9)	57 (60.0)	13 (13.7)	5 (5.3)	95 (100)

^a^Excerpts that described pharmacovigilance activities occurring within the country but that were not supported or funded by the current or past grants

^b^Excerpts that described pharmacovigilance activities that were previously funded by the grant-making organization but were not being supported in the current grant

### Qualitative findings

The primary PV-related focus area of grants was to improve drug quality monitoring, especially among PMI grants. These grants described activities to detect poor quality drugs along the supply chain and were linked to health systems strengthening activities as illustrated in the PMI 2016 grant to the Greater Mekong Sub-Region: *“PMI supports a broad array of health system strengthening activities which cut across intervention areas, such as training of health workers, supply chain management and health information systems strengthening, drug quality monitoring, and NCMP capacity building.”* These activities were often aimed at directly supporting the Ministry of Health in a given country to improve both forecasting the need for medications as well as ensuring their quality across PMI supported countries:

“PMI will continue to strengthen Ministry of Health in pharmaceutical management. PMI will provide technical assistance to the DNME, the Inspectorate General, and the NMCP in all steps of the procurement and distribution process: quantifying and forecasting demand of malaria commodities; procurement of quality malaria commodities; testing and quality assurance/quality control QA/QC of malaria commodities; planning distributions in accordance with needs; and distributing in an efficient and timely manner”

 (Angola, PMI 2016 Grant).

For many grants, drug quality assurance was just one aspect of a larger programme to improve supply chain management of malaria commodities. Multiple grants provide insufficient detail about these activities to fully categorize the activity as illustrated in the following quotes: “*Support quality assurance for antimalarials*.” (Thailand, Global Fund Grant), and *“PMI will also support the quality assurance of antimalarial products.”* (Liberia, PMI 2015 Grant).

PV-related activities were often bundled with activities intended to improve the regulatory capacity of the country to detect counterfeit drugs:

*“Improve drug regulatory capacity: Continue to support improvement of the regulatory and oversight capacities of the DNPL, revision of national list of essential drugs, and enhanced control of compliance to the pharmaceutical policy and regulations by PCG and the private pharmacies network. Capacity building of the pharmaceutical system will include improving capacity to combat counterfeit drugs and the illicit sale of drugs. ($250,000)”*

(Guinea PMI 2015 Grant).

These activities had a strong focus on capacity building and training staff in the Ministry of Health to improve implementation of malaria programmes.

Improving public health laboratories was a main focus of PV activities. Multiple grants, such as the Benin PMI 2015 grant, directly funded the purchase of Minilabs^®^, a product designed to combine the reagents and testing materials needed to perform basic tests to verify the quality of 80 essential medicines. In addition to provision of supplies, grants focused on capacity building staff and helping laboratories to achieve international accreditation:

“*Strengthen the national laboratory: Provide additional training and supervision to improve the functioning and quality of the national laboratory while maintaining the current MQM programme. Develop a five*-*year strategic plan outlining specific areas to strengthen, how to improve quality, and the sustainability of the laboratory in regards to maintaining staff, steps to take to achieve ISO 17025 accreditation, and improving the working relationship with the national regulatory authorities around counterfeit and sub*-*standard medicines. (Laboratoire National de Santé) ($100,000)”*

 (Mali, PMI 2016 Grant).

Twenty-two grants directly mentioned primaquine for malaria eradication in their country, and of these, ten grants funded PV and seven grants that funded these activities mentioned the need for systems to detect adverse event. This is highlighted in a planned evaluation in Ethiopia where PMI funded monitoring of the health effects after roll out of primaquine:

*“Evaluation of single*-*dose primaquine roll out ($500,000): This study will systematically monitor the effects of primaquine roll out on malaria transmission and reported drug toxicity and health costs through careful review of routinely collected malaria incidence data and cross sectional surveys to assess parasite burden and multiplicity of infection.”*

Similarly, in a 2015 grant to Senegal, PMI described supporting a *“detailed M&E plan, which will likely include regular supervision, data analysis, case investigation, and pharmacovigilance”* for implementation of low-dose primaquine in elimination districts.

No grant described a pregnancy exposure registry to detect adverse pregnancy outcomes after exposure to ACT in pregnancy. However, 84 (52.8%) grants directly funded malaria in pregnancy activities. The majority of these grants focused on prevention of malaria in pregnancy through procurement and distribution of LLINs and procurement and provision of IPT with sulfadoxine–pyrimethamine. Furthermore, 100 (62.9%) grants funded case management through drug procurement. In addition 58 (36.5%) grants directly provided funding for broad surveillance activities although these were not designed to specifically detect adverse events.

Of the 288 PV excerpts, 31 described non-specific PV and surveillance activities that were unfunded by the grant. For example the 2015 PMI grant for Liberia only stated “PMI will also support the quality assurance of anti-malarial products,” which does not provide any details on the types of activities to be funded. This lack of detail was also present in Global Fund grants as illustrated in a grant to Thailand, which only described “Support quality assurance for anti-malarials.”

In addition to multiple grants not providing details of the PV activities being funded, Global Fund grants contained standardized contractual language that described required PV activities despite PV activities not being directly funded in the budget. This scenario is best demonstrated by the following excerpt from a Pakistan Global Fund grant:

*“…the Principal Recipient shall ensure that random samples of Finished Pharmaceutical Products financed under the Agreement are obtained at different points in the supply chain, from initial receipt of the products in the Host Country to the delivery of those products to patients. Such samples shall be sent to one of the following laboratories for Quality Control testing…”*.

 These activities included ensuring drug quality throughout the supply chain within a country. In addition, the standardized language of the Global Fund grants also required countries to “*monitor adverse drug reactions according to existing international guidelines,”* as illustrated in a Global Fund grant to Tanzania.

## Discussion

Although this study identified 159 active malaria grants, only 37 (23%) grants directly funded PV-related activities in malaria-endemic countries, and no grants funded pregnancy exposure registries. Only 4.9% of grant excerpts described PV surveillance activities, such as detecting adverse events. The findings are similar to those of the assessment completed in 2010, which found that only 14 (34%) grants proposals requested funds for PV activities, and only one described a pregnancy registry [23]. The most common activities related to PV were laboratory support and training, improvement to the regulatory system, improving surveillance, and improving supply chain management. Grants were broadly focused on improvements to health systems instead of more narrowly focusing on improving the detection, assessment, understanding, and prevention of adverse events. These broad strategies to improve health systems can potentially be leveraged for PV activities, such as improved signal detection and signal evaluation.

Of the four main areas of safety concern for anti-malarials, the majority of funding has been focused on improving drug quality and preventing counterfeit anti-malarials from entering the market. The only specific PV activity funded by malaria grants was risk communication, often described as behaviour change communication in order to inform patients about the risks of counterfeit drugs. Given findings that up to one-third of all ACT has been previously reported as falsified or sub-standard in geographical areas within SSA and Southeast Asian countries [15], focusing on improving drug quality is a valuable activity for ensuring the success of malaria programmes.

This study found little evidence of funding for PV systems being requested for signal detection, signal evaluation, risk management, and risk communication. While no grants directly described PV systems conducting signal detection or signal evaluation, activities related to improvements to surveillance systems could be leveraged for signal detection and signal evaluation. Notably, this study found no evidence of support for pregnancy registries in low and middle-income countries. To alleviate this problem, the WHO has developed a protocol for implementing pregnancy exposure registries in LMIC which will be pooled into the WHO’s pregnancy registry database [26]. There may be opportunities for donors to partner with the WHO to build support for the implementation of the WHO’s protocol.

Despite a push towards malaria elimination in multiple countries, including the use of low-dose primaquine in conjunction with ACTs, only seven of 22 grants citing primaquine for eradication mentioned the additional need for PV systems to detect adverse events.

This study has some limitations. The study focuses on funding from Global Fund, PMI and the World Bank, and did not review grant materials from organizations such as the Bill & Melinda Gates Foundation, which does not make grant applications publically available, or the UK’s Department for International Development (DFID). The grants that were reviewed often provided very broad budget summaries, making it difficult to assess exactly how much money was being appropriated to PV activities. In addition, many grants provided few details on the exact activities planned, limiting the ability to categorize activities effectively. In addition, in 2011 the Global Fund changed their grant-making strategy from a round-based system to a system that provided funding in a more proactive, predictable and flexible manner [27]. This new funding system changed the grant application process, and newer grants provided fewer details on the intended use of grant funds.

The results of this analysis demonstrate that funding for PV has not been prioritized by key malaria donor organizations or by recipient countries, as reflected in their grant proposal submissions and MOPs. For the grants that designated funding for PV, the study found the emphasis was on drug quality assurance, not on adverse events. The current format of the grant proposals was insufficient for assessing the amount of funds appropriated and the activities to be conducted. Explicitly describing activities and providing more detailed budgets for grants is needed in order to fully access the sufficiency of the planned PV programme. Without future reformatting of grant-making guidelines and forms, it will continue to be difficult to determine trends over time, evaluate whether future investments provide sufficient resources for PV systems, and determine the effectiveness of such investments. Only one technical guidance document exists to assist countries when requesting financial support for PV in their proposals to the Global Fund [28]. There is a continued need for enhanced global guidance and technical assistance to help address these shortcomings and support country and donor goals of ensuring access to high quality and safe treatments for malaria and other conditions.

## Conclusions

Across 159 active Global Fund, PMI and World Bank grants for malaria, this study found only 37 (23%) grants directly funded PV activities in their budgets despite the recognition in the global health community that PV is a key component of any health system. Furthermore, only 4.9% of excerpts described activities regarding adverse event detection. This demonstrates a lack of appropriate emphasis placed on PV, despite the growth in the use of ACT and other anti-malarial courses of treatments provided in malaria-endemic countries. There was a great diversity in activities defined as related to PV, but very few grants focused on areas of PV such as surveillance systems for detecting and preventing adverse events, and no grant funded pregnancy exposure registries. Instead, the majority of funded activities focused on broadly improving drug quality and detecting and preventing counterfeit anti-malarials. Improvements to PV systems require stakeholders to collaborate on better definitions and agreeing to performance indicators. These efforts should inform where important gaps exist, allowing for donors and countries alike to contribute towards improving the safety of medicines. Investing in PV systems should benefit malaria control and prevention programmes and help ensure that robust laboratory and surveillance systems are in place for monitoring the safety and quality all essential medications.

## Additional file

**Additional file 1.** Pharmacovigilance quantitative methods and codes.
