# Supplementary material for: Donor support for quality assurance and pharmacovigilance of anti-malarials in malaria-endemic countries
Source: Malar J. 2017 Jul 11;16:282. doi: 10.1186/s12936-017-1921-x (PMC5504670; doi:10.1186/s12936-017-1921-x)
Supplement: Supplementary file 1 — Additional file 1. Pharmacovigilance quantitative methods and codes. [file 12936_2017_1921_MOESM1_ESM.docx]

**Pharmacovigilance Quantitative Methods and Codes**

Inclusion criteria for qualitative data:

Grant mentions at least one of the following:

1. Pharmacovigilance in the key word search

OR

1. Pharmacovigilance activities identified in the budget

OR

1. Keyword search identified the following words
   1. Counterfeit
   2. Substandard
   3. Falsified
   4. Adverse drug (>1 mention because it is included in the base grant language)
   5. Mass drug administration

Total number of included grants=57/159 reviewed

**A Priori Codes**: These codes will be used and added to as necessary. For each code, the intentionality of the activity will be measured on a 3-point likert scale (1 coincidental-3 intentional). For example an activity that is coincidently a HSS activity will be given a score of 1 and an activity that clearly states the objective is to design a new surveillance system will be given a score of 3.

1. **Pharmacovigilance**: Pharmacovigilance (PV) is defined as the science and activities relating to the detection, assessment, understanding and prevention of adverse effects or any other drug-related problem (WHO).
   1. Detecting and preventing counterfeit drugs
   2. Detecting low quality/substandard drugs
   3. Identifying adverse events in a non clinical trial environment including AE reporting or surveillance for AEs
2. **Supply Chain Management**: Movement and storage of pharmaceuticals from point of origin to point of use/care
   1. Stockouts
   2. Appropriate/Adequate storage and transport
   3. Long term planning and forecasting need
3. **Mass Drug Administration**: Mass drug administration conducted at a community or regional level whereby people receive drugs without formal diagnosis for treatment
4. **Public Health Surveillance**: the ongoing systematic collection, analysis, and interpretation of data, closely integrated with the timely dissemination of these data to those responsible for preventing and controlling disease and injury ([Thacker and Berkelman 1988](http://www.ncbi.nlm.nih.gov/books/NBK11770/)).
   1. Development of a new system (humans)
   2. Improvement of an existing system (humans)
   3. Conducting a survey (cross-sectional)
   4. Entomology survey
   5. Data analysis of HMIS data for surveillance purposes
5. **Health System Strengthening:** (i) the process of identifying and implementing the changes in policy and practice in a country’s health system, so that the country can respond better to its health and health system challenges[22](http://www.who.int/healthsystems/hss_glossary/en/22); (ii) any array of initiatives and strategies that improves one or more of the functions of the health system and that leads to better health through improvements in access, coverage, quality, or efficiency (WHO)
   1. Financial risk protection;
   2. Resource tracking
   3. Performance-based incentives
   4. Health governance;
   5. Costing and sustainability planning
   6. Human resources for health;
   7. Capacity building
   8. Monitoring and Evaluation (USAID)
      1. Monitoring outcome of a project
      2. Evaluation of projects’ performance
